# 839. Examining the Effects of HIV Infection on Severity of Outcomes in Those with COVID-19

**DOI:** 10.1093/ofid/ofab466.1035

**Published:** 2021-12-04

**Authors:** Melissa Parkinson, Rebecca Gerrity, Rachel Strength, Christian J Fuchs, Christopher Jackson, Dipen Kadaria, Ankur Seth, Chinelo N Animalu, Nathan A Summers

**Affiliations:** University of Tennessee Health Science Center, Memphis, Tennessee

## Abstract

**Background:**

Throughout the SARS-CoV-2 pandemic, there have been many questions about how COVID-19 affects patients living with HIV (PLWH). We examined the clinical courses of 45 PLWH who required hospitalization with SARS-CoV-2 infection.

**Methods:**

This is a retrospective cohort study in which ICD-10 codes were used to identify PLWH who were admitted to three large hospital systems in Memphis, TN with COVID-19. We included all patients ≥ 18 years of age with HIV and a documented positive SARS-CoV-2 PCR test. After manual abstraction from the electronic health records, chi-squared and T-tests were performed to evaluate associations between patient-level factors and outcomes.

**Results:**

A total of 45 patients with HIV who tested positive for SARS-CoV-2 were admitted to Memphis, TN area hospitals between March 2020 and October 2020. 18 (40%) were female, 43 (95.6%) were Black, and the average age was 50.3 years (SD 12.6). The average BMI was 30.2 (SD 8.6). 40 (88.9%) patients admitted had at least one comorbidity with the most common being hypertension (28 patients, 62.2%) and diabetes (14 patients, 31.1%). 24 (46.7%) patients had a Charlson Comorbidity Index > 3. 15/43 (48.4%) patients had a CD4 count < 200, and 35 (77.8%) were on ART. 30 (66.7%) patients met SIRS criteria within 24 hours of admission, and 27 (60%) required some form of oxygen supplementation during hospitalization, including 4 (8.9%) who required intubation. The average length of stay was 10.4 days (SD 12.5). 9 (20%) patients required an ICU stay, and 3 (6.7%) died. BMI > 30, CD4 count < 200, and viral load > 1000 were not associated with worse outcomes. Both a Charlson Comorbidity Index > 3 and the absence of ART were associated with need for ICU-level care.

**Conclusion:**

Viral load, CD4 count, and BMI were not correlated with differences in mortality or oxygen use in our study. Patients with higher Charlson Comorbidity Indices and patients who were not on ART at presentation were significantly more likely to require the ICU. Further study is needed to definitively determine factors affecting the outcomes of PLWH with SARS-CoV-2 infection.

**Disclosures:**

**All Authors**: No reported disclosures

